# Alleviation effects of epigallocatechin-3-gallate against acute kidney injury following severe burns

**DOI:** 10.1007/s10157-023-02414-1

**Published:** 2023-10-17

**Authors:** Hongyan Xu, Yichao Pang, Wei Sun, Yi Luo

**Affiliations:** 1 Yangzhou University, Yangzhou City, 225009 Jiangsu Province China; 2 Clinical Medical College, Yangzhou University, Yangzhou City, 225001 Jiangsu Province China

**Keywords:** Burns, Acute kidney injury, EGCG, Inflammation, Oxidative stress

## Abstract

**Background:**

Burn patients often face a high risk of acute kidney injury (AKI) after severe burn injuries, meanwhile epigallocatechin-3-gallate (EGCG) has been proven to be effective in alleviating organ injury.

**Methods:**

This study used the classical burn model in rats. Thirty model rats were randomly divided into a Burn group, a Burn + placebo group, a Burn + EGCG (50 mg/kg) group, and ten non-model rats as Sham group. The urinary excretion of the rats was subsequently monitored for a period of 48 h. After 48 h of different treatments, rat serum and kidneys were taken for the further verification. The efficacy of EGCG was assessed in pathological sections, biochemical indexes, and at the molecular level.

**Results:**

Pathological sections were compared between the Burn group and Burn + placebo group. The rats in the Burn + EGCG group had less kidney damage. Moreover, the EGCG group maintained significantly elevated urine volumes, biochemical indexes manifested that EGCG could reduce serum creatinine (Cr) and neutrophil gelatinase-associated lipocalin (NGAL) level and inhibit the oxidation-related enzyme malondialdehyde (MDA) level, meanwhile the superoxide dismutase (SOD) level was increased. The molecular level showed that EGCG significantly reduced the mRNA expression levels of the inflammation-related molecules interleukin-6 (IL-6) and tumor necrosis factor-α (TNF-α).

**Conclusion:**

The research indicated that EGCG had an alleviating effect on kidney injury in severely burned rats, and its alleviating effects were related to improving kidney functions, alleviating oxidative stress, and inhibiting the expression of inflammatory factors.

## Introduction

Burns occur frequently in the population due to accidental injuries. These first cause thermodynamic damage to the skin and then induce multiple organ damage [1]. Massive fluid is lost due to the skin defects in the early stages of burn injuries, resulting in insufficient blood volume in the body. Consequently, with intravascular hypovolemia, the kidney becomes vulnerable as one of the organs with the most abundant blood supply. Equally, oxidative stress and inflammation participates in the progression of kidney injury [2–4]. Severe burn injuries can lead to the development of acute kidney injury (AKI), significantly increasing the risk of mortality, which ranged from 28 to 100% [5–7]. AKI is defined within 48 h attenuate in kidney function, which encompasses acute kidney failure and other complications. Clinically, AKI is a syndrome that has various etiologies caused by the presence of the postoperative, severe trauma, infection, nephrotoxic drugs, and so on [8, 9]. The onset of AKI can be attributed to various factors following severe burns, such as hypovolemia, a robust inflammatory response, excessive accumulation of denatured proteins, sepsis, and severe organic dysfunction [10]. Prompt recognition and timely intervention are crucial measures for improving the prognosis of patients with burn-related AKI.

Herbal or medicinal plant-derived natural compounds including tea are extensively utilized for the treatment and prevention of various ailments. EGCG is the most abundant and bioactive polyphenol catechin in green tea, its molecular formula is C_22_H_18_O_11_ [11]. Numerous studies, both in vitro and in vivo, have explored the health benefits of green tea and its main chemical component EGCG in various human diseases, including kidney disease, EGCG has potential therapeutic and preventive effects on a variety of illnesses and diseases, these included cancer, obesity, cardiovascular disease, liver disease, nerve injury and degeneration diseases, and immune diseases [11–18]. Besides, it is worth mentioning that EGCG may have positive significance in the treatment of COVID-19 [19, 20]. The anti-oxidative, anti-inflammatory, and anti-apoptotic properties of EGCG have broad application prospects for its use as an alternative strategy to treat or prevent various kidney diseases, including kidney stone disease, cisplatin-induced nephrotoxicity, glomerulonephritis, renal cell carcinoma, lupus nephritis, diabetic nephropathy, chronic kidney disease, and renal fibrosis [21].

Although a large body of studies has proven the health benefits of EGCG for its anti-oxidative, anti-inflammatory, and anti-apoptotic properties, there is currently insufficient evidence in the literature to support the use of EGCG in treating AKI resulting from severe burns. Therefore, this study is the first to evaluate the therapeutic effect of EGCG on AKI in rats with severe burns. It is focused on various discovered potential effects of EGCG that provides new ideas for the future clinical adjuvant treatment of AKI which are induced by severe burns.

## Materials and methods

### Animals

A total of 40 male Sprague–Dawley (SD) rats (250–260 g) were purchased from Yangzhou University (Jiangsu, China). They were raised in a 12 h light/12 h dark cycle house with positive pressure sterilizing and ventilation functions, with a controlled normal temperature (21–25 °C) and humidity, normal food and drink. All experimental protocols on animals had been approved by the committee on animal care of Yangzhou University.

### The severe burn model

After adaptive feeding of the SD rats for 7 days, the classical rat burn model was constructed according to the method proposed by Feng et al. and Guo et al. [22, 23]. Each rat was anesthetized with a dose of 1% pentobarbital sodium (50 mg/kg, intraperitoneal injection), and about 40% of the total body surface area (TBSA) was shaved from their backs. The experimental animals were bound with bandages and the exposed area of the rats’ back was horizontally immersed in 95 °C boiling water for 15 s, resulting in 2nd degree burns.

### Drug treatment

Thirty model rats were randomly assigned to Burn group, Burn + placebo group (saline, 10 ml/kg) received an immediate intraperitoneal injection after burn, Burn + EGCG group (EGCG, 50 mg/kg) received an immediate intraperitoneal injection after burn [24, 25], and Burn + placebo group and burn + EGCG group shared an equivalent amount of the saline solution. Another 10 rats were exposed to 20 °C water for 15 s after the anesthesia as the sham group. All the rats were kept in individual cages and given a Lactate Ringer solution (LRS) at 4 ml/kg/TBSA for liquid resuscitation via intraperitoneal injections immediately after the operation. In addition, all the rats accepted a hypodermic injection of 0.25 mg/kg buprenorphine for analgesia immediately after the surgery, this was followed by maintenance analgesia treatment every 12 h.

### Urine collection and tissue preparation

An amount of pre-weighed sawdust was uniformly distributed in the cages, and the sawdust weight in each cage was measured every 6 h. The urine volume of the rats was determined by measuring the weight of the sawdust (1 g/mL).

All the rats were sacrificed 48 h after being burned. Blood was collected through a heart puncture of a general anesthesia (pentobarbital sodium, 50 mg/kg), and serum was obtained by centrifugation at 3000 rpm for 10 min at 4 °C, and stored at − 80 °C. Kidney tissues were dissected and removed. The left kidneys from all the rats were fixed in a 10% formalin solution for pathological evaluation; equally, the right kidneys were stored at − 80 °C for molecular biological detection and kidney tissue biochemical detection.

### Histopathological analysis

The fixed kidneys were cut into 6-μm sections thick. All the sections were stained with hematoxylin and eosin (HE) after being embedded in paraffin wax. Subsequently, the sections were observed under the microscope to observe the degree of kidney injury caused by severe burns.

### Kidney function analysis

Serum creatinine (Cr) was detected via kit (Nanjing Jiancheng Bioengineering Institution, China). The neutrophil gelatinase-associated lipocalin (NGAL) levels were measured with the neutrophil gelatinase-associated lipocalin assay kit (Nanjing Jiancheng Bioengineering Institution, China) according to the manufacturer’s instructions.

### Measurement of antioxidant enzymatic activity

The kidney tissues from the various groups were weighed, and 1 g of the kidney tissue was homogenized in 9 ml of precooled normal saline, followed by centrifugation. Malondialdehyde (MDA) levels and superoxide dismutase (SOD) activity from each supernatant were tested via the kits (Nanjing Jiancheng Bioengineering Institution, China) according to the manufacturer’s protocols.

### Immunohistochemistry (IHC) staining

Immunohistochemistry (IHC) staining paraffin-embedded (5-μm-thick slices) were examined by IHC and IF staining. Some sections were incubated with anti-interleukin-6 (IL-6) antibodies (Abcam, UK) overnight at 4 °C. And then the sections were incubated with goat anti-rabbit secondary antibody (Servicebio, China), and visualized with a 3,3-diaminobenzidine (DAB) kit (Servicebio, China). Finally, the mounted sections were observed and photographed under a microscope at 200** ×** magnification (E100, Nikon, Japan).

### Quantitative real-time PCR (qRT-PCR) analysis of inflammatory factors gene expressions

The expression levels of IL-6 and TNF-α were measured via qRT-PCR. The total RNA was extracted from the kidney tissues by Trizol (Invitrogen, USA), and Qubit 3.0 (Thermo Scientific, USA) was used in the quantification of RNA. RNA was reverse transcribed to cDNA using TransScript III First-Stand Synthesis SuperMix for qRT-PCR (Invitrogen, USA). The reaction system and procedure were applied according to the manufacturer’s instructions. The primers (Table 1) were designed by Shanghai Biological Engineering Co., Ltd. (Shanghai, China). Expression levels were assessed relative to the 18S rRNA, as an internal standard. The levels of the inflammatory factors gene expressions were expressed using the 2^−∆ ∆Ct^ method.

**Table 1:** Primer sequences

Gene	Primer sequences (5′–3′)
Rat IL-6	Forward: 5′-TGACAGCCACTGCCTTCCCTAC-3′
	Reverse: 5′-CAATCAGAATTGCCATTGCACAA-3′
Rat TNF-α	Forward: 5′-GCCACCACGCTCTTCTGTCTACTG-3′
	Reverse: 5′-TGGGCTACGGGCTTGTCACTC-3′
Rat 18S	Forward: 5′-GAATTCCCAGTAAGTGCGGGTCATA-3′
	Reverse: 5′-CGAGGGCCTCACTAAACCATC-3′

### Statistical analysis

The Statistical Product and Service Solutions (SPSS) version 23.0 (IBM Corporation, USA) software was used for the statistical analysis. The results were presented as the mean ± standard (SD). Comparisons between the groups were conducted using Duncan multiple comparison test. Significance was accepted at a value of *P* < 0.05.

## Results

### EGCG increased the urine volume in the severely burned rats

**Fig. 1:**
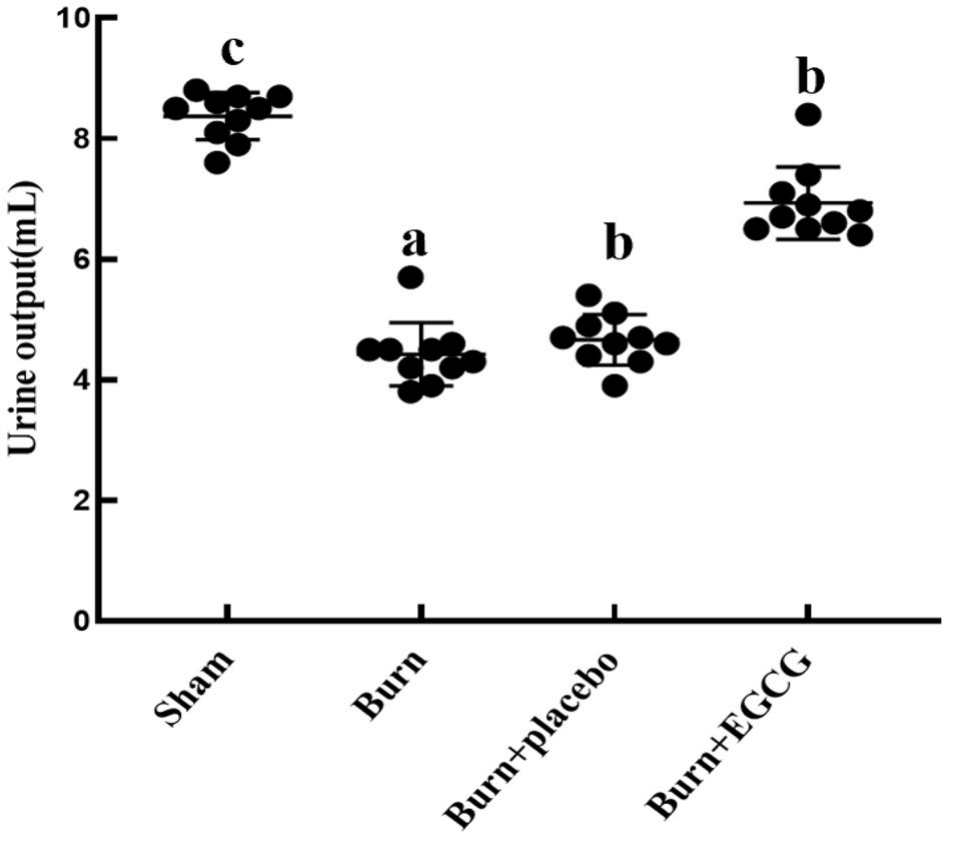
Urine volume of all the experiments groups. The sample size was *n* = 10 for each group. The results were expressed as the means ± SD. Means in the same biochemical test indicator with the different letter are significantly different (*P* < 0.05)

We estimated the kidney function in the rats by the urine output, which is shown in Fig. 1. The urine output of rats in the EGCG, Burn, and Burn + placebo groups was significantly decreased compared to that of the Sham group after severe burn insult (*P* < 0.05). However, the EGCG group maintained a significantly elevated urine volume compared to both the Burn and Burn + placebo groups (*P* < 0.05).

### EGCG weakened the severity of kidney tubular damage in severely burned rats

**Fig. 2:**
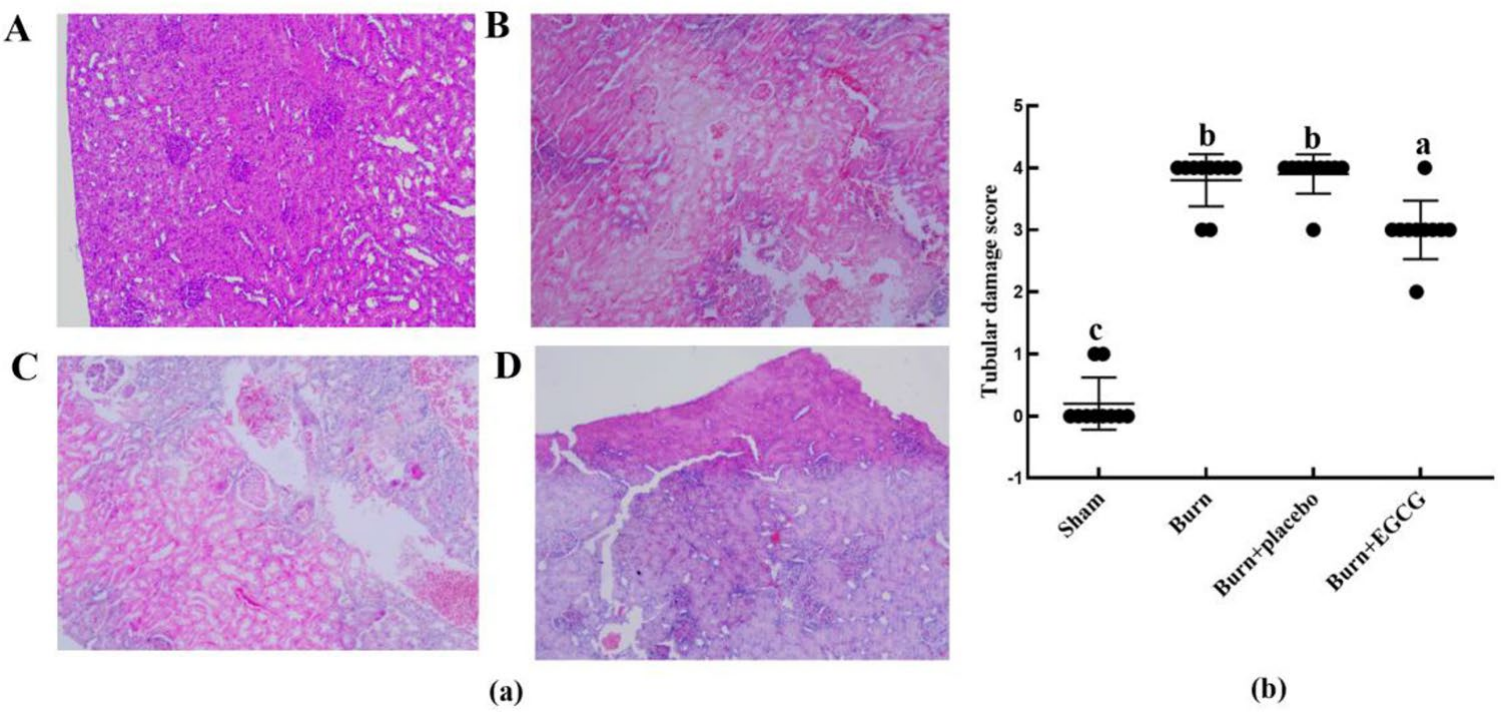
Histological evaluations of tubular damage in the early stage of post-burn. Representative HE-staining images of all the experiments groups manifested histological evidence of renal tubular damage at a magnification of × 100. Furthermore, the tubular damage scores provided quantitative verification (**b**)

The kidney damage in rats was analyzed via histological examination, which is shown in Fig. 2. The structure of kidney tubular in the sham group was normal. The scores of the tubular damage showed a significant increase in rats at 48 h after burn (*P* < 0.05). However, the tubular damage scores were markedly reduced in the Burn + EGCG group of rats compared to those in the Burn and Burn + placebo groups (*P* < 0.05).

### EGCG decreased the levels of serum Cr and NGAL

**Fig. 3:**
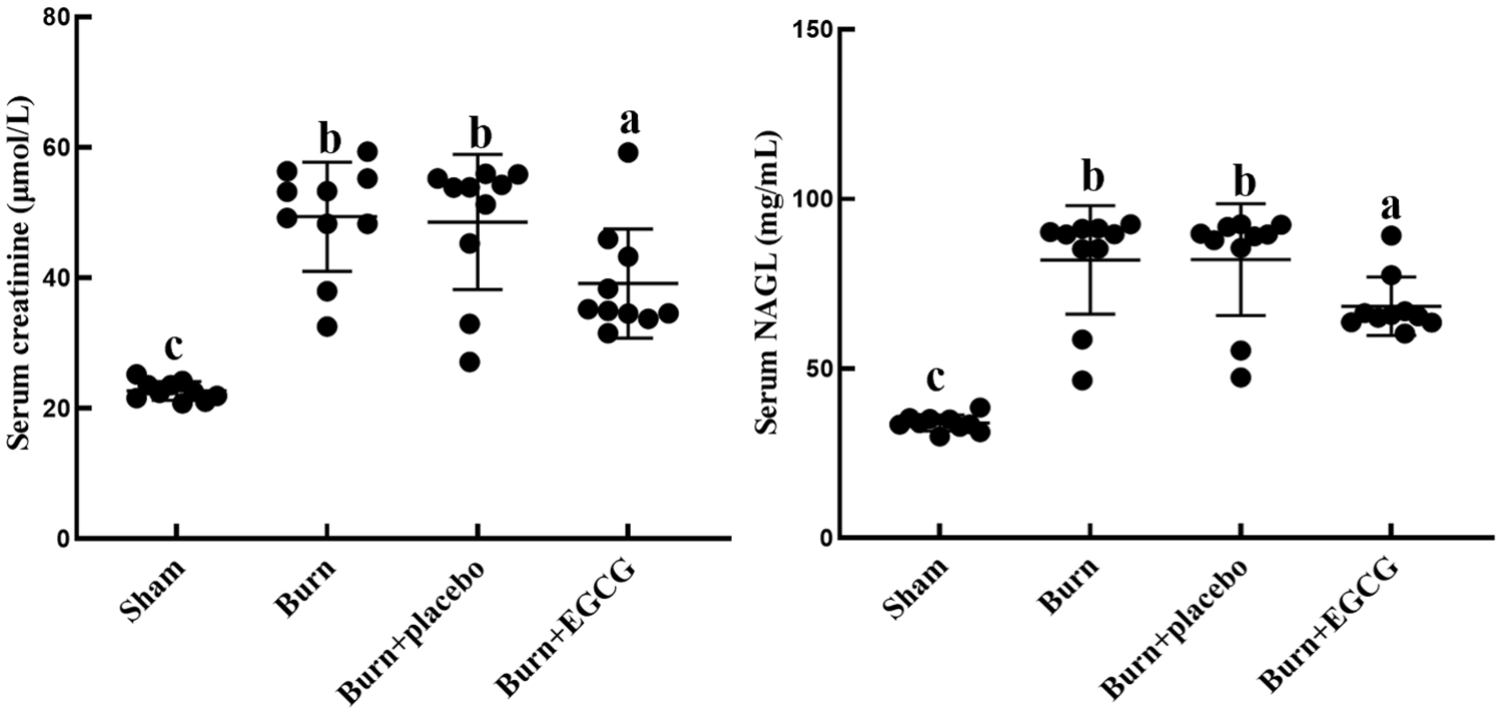
Serum measurements of creatinine (μmol/L) and NAGL (mg/mL) levels of all the experiments groups. The sample size was *n* = 10 for each group. The results were expressed as the means ± SD. Means in the same biochemical test indicator with the different letter are significantly different (*P* < 0.05)

We investigated the levels of serum Cr and NGAL in all the groups after the severe burn injuries as shown in Fig. 3. The Burn group had markedly clear raised elevation levels of the serum Cr and NGAL values in their kidney tissues compared with the Sham group (*P* < 0.05). Inversely, EGCG treatment clearly displayed significant reductions in the serum Cr and NGAL levels in the Burn + EGCG group compared with the Burn group and Burn + placebo group (*P* < 0.05), despite the serum Cr and NGAL levels of Burn + EGCG group still being higher than the Sham group.

### EGCG attenuates oxidative stress in the kidney tissues of the severely burned rats

**Fig. 4:**
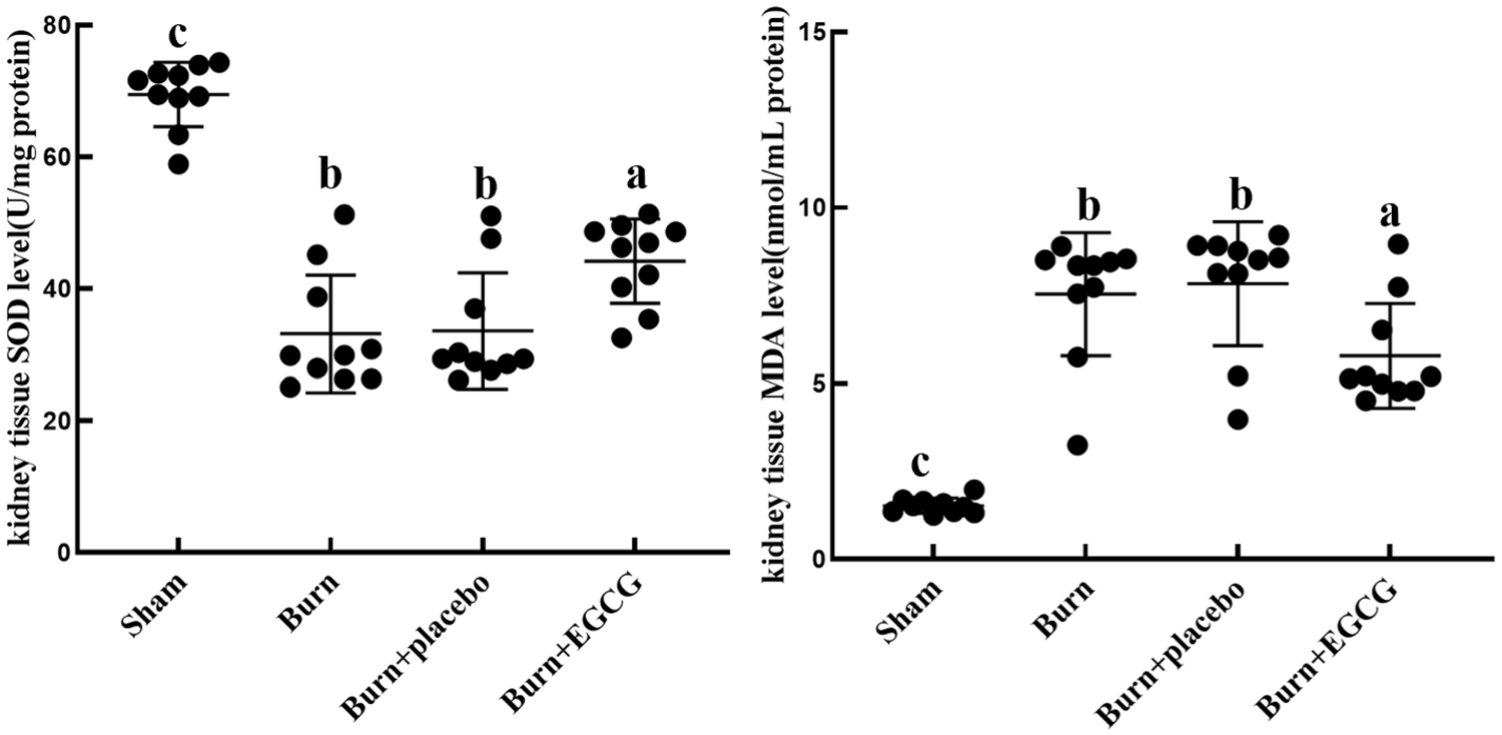
The effect of EGCG on kidney tissue SOD (U/mg protein) levels and MDA (nmol/mL protein) levels. The sample size was *n* = 10 for each group. The results were expressed as the means ± SD. The means in the same biochemical test indicator with a different letter are significantly different (*P* < 0.05)

The activity changes of the endogenous antioxidant enzymes SOD and MDA are shown in the Fig. 4, severe burn induced a sharp increase in the MDA levels (*P* < 0.05), and in addition, the levels of SOD were significantly decreased in the Burn group. However, EGCG treatment significantly reduced the levels of MDA and brought a rise in SOD activities in comparison with the Burn group and the Burn + placebo group (*P* < 0.05).

### EGCG makes changes of IL-6 and inhibits the expression of inflammatory factors in the kidney tissues of severely burned rats

**Fig. 5:**
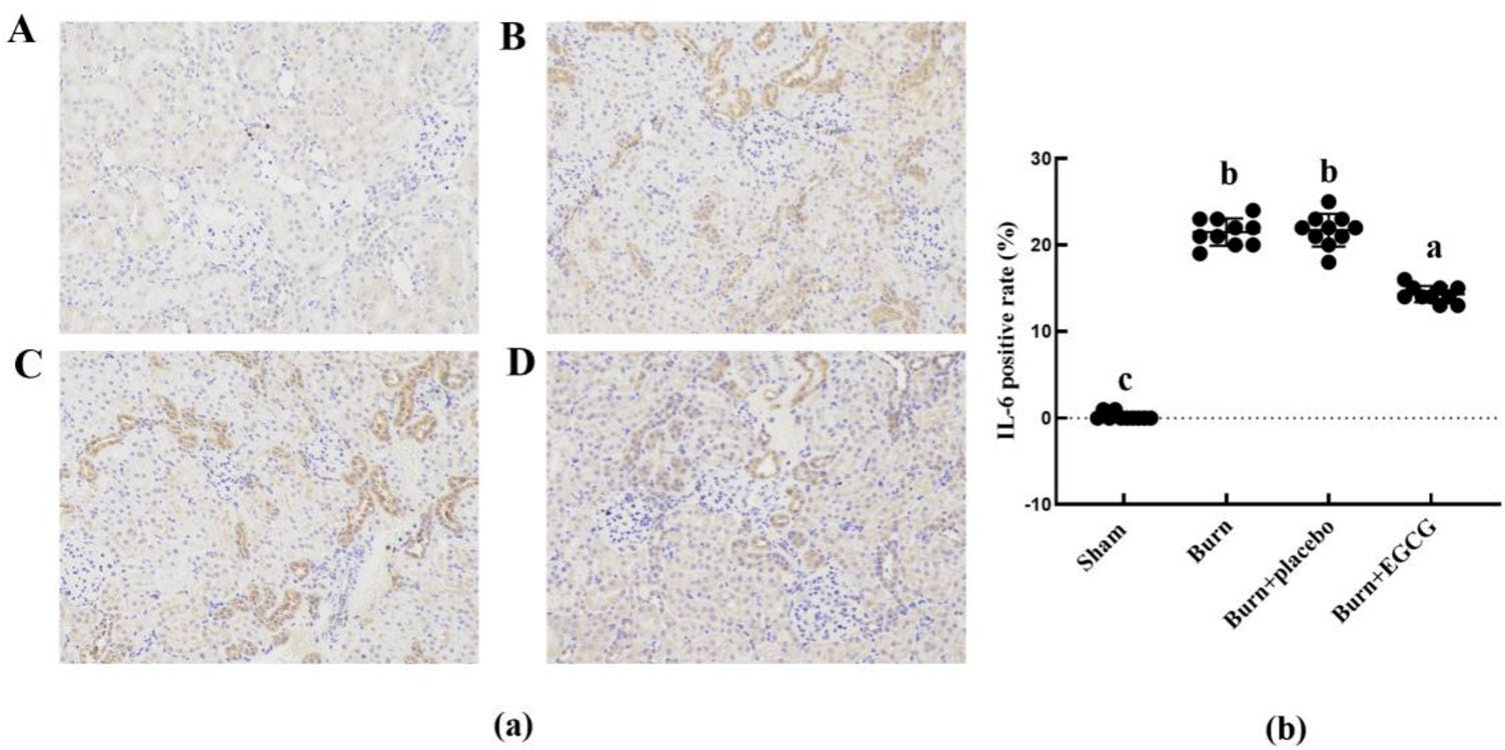
Immunohistochemistry renal tissue staining in renal tissue. The sample size was *n* = 10 for each group. The results were expressed as the means ± SD. Means in the same biochemical test indicators with the different letters are significantly different (*P* < 0.05)

IL-6 was detected via IHC staining in the kidney tissues after the severe burn injury as shown in Fig. 5, the severe burn injury was related to increased numbers of the positively labeled cells in the rat kidneys (*P* < 0.05). Following EGCG treatment, these burn-induced IL-6 level rises were gradually lowered compared with the Burn group and the Burn + placebo group (*P* < 0.05).

**Fig. 6:**
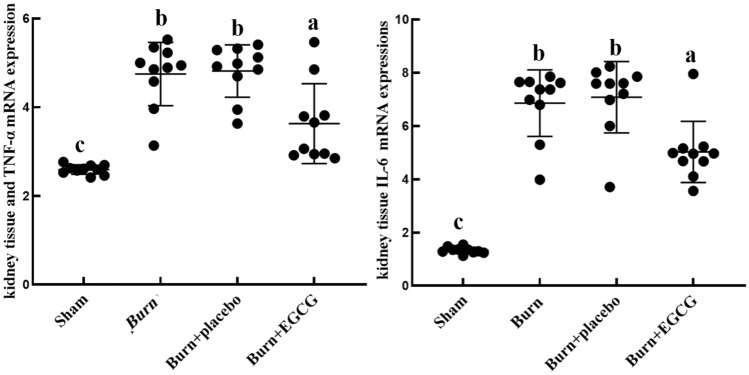
Analysis of the kidney tissue IL-6 and TNF-α mRNA expressions of all the groups via quantitative RT-PCR. The results demonstrated that EGCG treatment could significantly decrease the expression of the inflammatory factors in rat kidneys after the body burns. The sample size was *n* = 10 for each group. The results were expressed as the means ± SD. Means in the same biochemical test indicators with the different letters are significantly different (*P* < 0.05)

We evaluated the severe burn-induced inflammation by detecting the IL-6 and TNF-α mRNA expression in the kidney tissues of the rats using quantitative RT-PCR, as shown in Fig. 6. The expressions of the IL-6 and TNF-α in the Burn group were significantly elevated after the burn injury (*P* < 0.05). EGCG led to a markedly decreased kidney tissues IL-6 and TNF-α mRNA expression in the Burn + EGCG group.

## Discussion

In this study, based on the reported potential role of EGCG, we have recently revealed the potential protective role of EGCG in AKI after severe burns in rats from various perspectives. The experimental results concluded that EGCG provided a potential therapeutic value against AKI following severe burns. The potential clinical use of EGCG was associated with anti-oxidative, anti-inflammatory effects, meanwhile EGCG improved kidney function.

In our experiment, the classical rat burn model was built using the hot water bath, which tries to mimic the physiological conditions post-burn. In addition, we eliminated the effect of the hypovolemic shock on kidney tissues by fluid replacement treatment. The changes in histological structure and function were more distinct at 48 h after the severe burns, which might be the best time window for observation according to a previous report [26]. First, we evaluated whether EGCG could alleviate early renal injury after burn in rats from two aspects: histopathology and functional indicators. According to previous literature, researchers have also discovered that AKI can lead to tubular necrosis, which subsequently impairs renal function [27, 28]. The experiment showed that EGCG treatment significantly attenuated the renal tubule injury scores of burned rats, indicating its ability to alleviate structural damage in burned renal tissue. In subsequent functional evaluations, EGCG was found to reduce sensitivity indicators associated with acute kidney injury following burns. Although evaluation criteria for the clinical assessment of AKI are inconsistent, Kidney Disease: Improving Global Outcomes (KDIGO) clinical practice guidelines and the Risk-Injury-Failure-Loss-End (RIFLE) system was widely acknowledged. This states that urinary output criteria and serum Cr are the key factors in evaluating AKI. This includes changes in serum Cr (≥ 26.5 μmol/l or 0.3 mg/dl) at 48 h [8, 29]. Besides, reduced circulatory volume and tubular necrosis were responsible for the decrease of the glomerular filtration rate (GFR), leading to reduced urine volumes and it accelerated the progression of impaired renal function [10]. In addition, present studies have shown that the biomarkers of kidney cell injury, NGAL may sensitively identify AKI at an earlier stage [30]. In our study, EGCG ameliorated the reduction in urine volume and significantly attenuated the level of the serum Cr and NGAL. In summary, EGCG can alleviate the process of AKI in rats after burns, and in the follow-up study, we discussed the mechanism of kidney protection provided by EGCG.

Next, the body produces excessive amounts of reactive oxygen species (ROS) and reactive nitrogen under the stimulus of trauma or disease, which exceed the scavenging activity of the tissue cells, resulting in oxidative stress [31]. ROS can be generated in the body, leading to lipid peroxidation of structures rich in lipids such as cell membranes and the production of harmful substances like MDA. Conversely, ROS-mediated oxidative stress reactions can trigger a significant increase in endogenous antioxidant enzymes such as SOD, resulting in their depletion and compromising the body’s natural defense against external stimuli. In this study, EGCG significantly reduced MDA levels, indicating that EGCG alleviates oxidative stress through its anti-lipid peroxidation. At the same time, there are various antioxidant enzymes such as SOD in the body. We have also observed that EGCG effectively enhances the activity of SOD, suggesting its potential to restore endogenous antioxidant enzyme activity. These findings align with previous research studies, Kosar Kiai et al. detected that EGCG may be effective against neurodegeneration by increasing the activity of SOD and decreasing the activity of MDA [32]. In this study, EGCG can alleviate kidney tissue oxidative stress injury caused by burns by scavenging ROS, inhibiting lipid peroxidation induced by ROS, and restoring the activity of endogenous antioxidant enzymes. Palabiyik et al. detected that EGCG treatment significantly improved the oxidative stress induced by contrast-induced nephrotoxicity (CIN) [25]. However, there exists substantial distinctions in both the clinical characteristics and pathophysiological mechanisms between CIN and burn-induced AKI. The etiology of early AKI related to burns involves hypovolemia resulting from significant fluid loss, elevated levels of inflammatory mediators, and the release of denatured proteins; besides, CIN is caused by direct tubular toxicity and contrast media-induced reduction of renal perfusion [10, 33]. Therefore, it is worth further exploration of the distinct molecular pathways through which EGCG exerts its antioxidant function in these two contexts.

Hence, inflammation is also one of the main causative factors of organ injury following burns. Moreover, it has been reported that the release of inflammatory mediators might eventually cause tubular damage [34]. Generally, immune cells secrete anti-inflammatory cytokines and enzymes such as IL-6, TNF-α, IL-1β, IL-4, and cyclooxygenases (COXs) after burn injuries to intervene in the process of organ damage [35, 36]. According to the results of this experiment, the expressions of inflammatory factors IL-6 and TNF-α were significantly increased, indicating the strong response to injurious stimuli. Tomokazu Ohishi et al. also reported that EGCG bring a marked decrease in inflammatory factor TNF-α and IL-6 levels to attenuate inflammation [37]. In this study, EGCG also showed an effective effect in alleviating the release of inflammatory mediators in kidney tissue induced by burns. Immunohistochemical staining also showed that EGCG could reduce the expression distribution of inflammatory factor IL-6 in the kidney tissue of burned rats. Finally, it is suggested that it may alleviate acute kidney injury after burns in rats through an anti-inflammatory effect.

Although this study has verified the potential protective effect of AKI on acute kidney injury following burn injuries and preliminarily discussed the potential mechanism involved in its protective effect on the kidney, it is still unclear which signaling pathway is regulated by AKI at the molecular level to achieve these effects, and we will further explore it.

## Conclusion

In summary, the previous study first illuminated the potential intervention of EGCG against AKI in rats after burn injuries. The protective ability of the EGCG is associated with improving kidney functions, relieve oxidant stress and inflammation.

## References

[1] Hoesel LM (2009). Local wound p38 MAPK inhibition attenuates burn-induced cardiac dysfunction. Surgery.

[2] Li H (2014). Lithium chloride suppresses colorectal cancer cell survival and proliferation through ROS/GSK-3beta/NF-kappaB signaling pathway. Oxid Med Cell Longev.

[3] Zhao L (2013). Protective effect of hydrogen-rich saline on ischemia/reperfusion injury in rat skin flap. J Zhejiang Univ Sci B.

[4] Moreira E, Burghi G, Manzanares W (2018). Update on metabolism and nutrition therapy in critically ill burn patients. Med Intensiva (Engl Ed).

[5] Witkowski W (2016). Early and late acute kidney injury in severely burned patients. Med Sci Monit.

[6] Coca SG (2007). Contribution of acute kidney injury toward morbidity and mortality in burns: a contemporary analysis. Am J Kidney Dis.

[7] Dépret F (2018). Prediction of major adverse kidney events in critically ill burn patients. Burns.

[8] Kellum JA, Lameire N, K.A.G.W. Group (2013). Diagnosis, evaluation, and management of acute kidney injury: a KDIGO summary (Part 1). Crit Care.

[9] Makris K, Spanou L (2016). Acute kidney injury: definition, pathophysiology and clinical phenotypes. Clin Biochem Rev.

[10] Niculae A (2022). Burn-induced acute kidney injury-two-lane road: from molecular to clinical aspects. Int J Mol Sci.

[11] Zeng L, Holly JM, Perks CM (2014). Effects of physiological levels of the green tea extract epigallocatechin-3-gallate on breast cancer cells. Front Endocrinol (Lausanne).

[12] Suganuma M (2020). Stiffening of cancer cell membranes is a key biophysical mechanism of primary and tertiary cancer prevention with green tea polyphenols. Chem Pharm Bull (Tokyo).

[13] Choi C (2020). Epigallocatechin-3-gallate reduces visceral adiposity partly through the regulation of beclin1-dependent autophagy in white adipose tissues. Nutrients.

[14] Zhang C (2020). Exosomes derived from epigallocatechin gallate-treated cardiomyocytes attenuated acute myocardial infarction by modulating microRNA-30a. Front Pharmacol.

[15] Naito Y (2020). Epigallocatechin-3-gallate (EGCG) attenuates non-alcoholic fatty liver disease via modulating the interaction between gut microbiota and bile acids. J Clin Biochem Nutr.

[16] Park DJ, Kang JB, Koh PO (2020). Epigallocatechin gallate alleviates neuronal cell damage against focal cerebral ischemia in rats. J Vet Med Sci.

[17] Pervin M (2018). Beneficial effects of green tea catechins on neurodegenerative diseases. Molecules.

[18] Leong DJ (2014). Green tea polyphenol treatment is chondroprotective, anti-inflammatory and palliative in a mouse post-traumatic osteoarthritis model. Arthritis Res Ther.

[19] Menegazzi M (2020). Protective effect of epigallocatechin-3-gallate (EGCG) in diseases with uncontrolled immune activation: could such a scenario be helpful to counteract COVID-19?. Int J Mol Sci.

[20] Jang M (2020). Tea polyphenols EGCG and theaflavin inhibit the activity of SARS-CoV-2 3CL-protease in vitro. Evid Based Complement Altern Med.

[21] Kanlaya R, Thongboonkerd V (2019). Protective effects of epigallocatechin-3-gallate from green tea in various kidney diseases. Adv Nutr.

[22] Feng Y (2013). Sustained oxidative stress causes late acute renal failure via duplex regulation on p38 MAPK and Akt phosphorylation in severely burned rats. PLoS ONE.

[23] Guo SX (2015). Effects of hydrogen-rich saline on early acute kidney injury in severely burned rats by suppressing oxidative stress induced apoptosis and inflammation. J Transl Med.

[24] Zhang F (2015). Neuroprotective effects of (–)-epigallocatechin-3-gallate against focal cerebral ischemia/reperfusion injury in rats through attenuation of inflammation. Neurochem Res.

[25] Palabiyik SS (2017). A new update for radiocontrast-induced nephropathy aggravated with glycerol in rats: the protective potential of epigallocatechin-3-gallate. Ren Fail.

[26] Guo SX. Benefical effect of astaanthin on early acute kidney injury in severely-burned rats. Zhejiang University. 2017.

[27] Homma K (2015). Inhalation of hydrogen gas is beneficial for preventing contrast-induced acute kidney injury in rats. Nephron Exp Nephrol.

[28] Merter AA (2015). Protective effects of amifostine on ischemia-reperfusion injury of rat kidneys. Indian J Pharmacol.

[29] Mehta RL (2007). Acute kidney injury network: report of an initiative to improve outcomes in acute kidney injury. Crit Care.

[30] Albert C (2020). Neutrophil gelatinase-associated lipocalin measured on clinical laboratory platforms for the prediction of acute kidney injury and the associated need for dialysis therapy: a systematic review and meta-analysis. Am J Kidney Dis.

[31] Li Q (2021). Nanozymes regulate redox homeostasis in ROS-related inflammation. Front Chem.

[32] Kian K (2019). Neuroprotective effects of (-)-epigallocatechin-3-gallate (EGCG) against peripheral nerve transection-induced apoptosis. Nutr Neurosci.

[33] Feldkamp T, Kribben A (2008). Contrast media induced nephropathy: definition, incidence, outcome, pathophysiology, risk factors and prevention. Minerva Med.

[34] Korkmaz A, Kolankaya D (2010). Protective effect of rutin on the ischemia/reperfusion induced damage in rat kidney. J Surg Res.

[35] Pollard JW (2004). Tumour-educated macrophages promote tumour progression and metastasis. Nat Rev Cancer.

[36] Minciullo PL (2016). Inflammaging and anti-inflammaging: the role of cytokines in extreme longevity. Arch Immunol Ther Exp (Warsz).

[37] Ohishi T (2016). Anti-inflammatory action of green tea. Antiinflamm Antiallergy Agents Med Chem.

